# It’s positive to be negative: Achilles tendon work loops during human locomotion

**DOI:** 10.1371/journal.pone.0179976

**Published:** 2017-07-03

**Authors:** Karl E. Zelik, Jason R. Franz

**Affiliations:** 1 Department of Mechanical Engineering, Vanderbilt University, Nashville, TN, United States of America; 2 Department of Biomedical Engineering, Vanderbilt University, Nashville, TN, United States of America; 3 Department of Physical Medicine & Rehabilitation, Vanderbilt University, Nashville, TN, United States of America; 4 Joint Department of Biomedical Engineering, University of North Carolina and North Carolina State University, Chapel Hill, NC, United States of America; Northwestern University, UNITED STATES

## Abstract

Ultrasound imaging is increasingly used with motion and force data to quantify tendon dynamics during human movement. Frequently, tendon dynamics are estimated indirectly from muscle fascicle kinematics (by subtracting muscle from muscle-tendon unit length), but there is mounting evidence that this Indirect approach yields implausible tendon work loops. Since tendons are passive viscoelastic structures, when they undergo a loading-unloading cycle they must exhibit a negative work loop (i.e., perform net negative work). However, prior studies using this Indirect approach report large positive work loops, often estimating that tendons return 2–5 J of elastic energy for every 1 J of energy stored. More direct ultrasound estimates of tendon kinematics have emerged that quantify tendon elongations by tracking either the muscle-tendon junction or localized tendon tissue. However, it is unclear if these yield more plausible estimates of tendon dynamics. Our objective was to compute tendon work loops and hysteresis losses using these two Direct tendon kinematics estimates during human walking. We found that Direct estimates generally resulted in negative work loops, with average tendon hysteresis losses of 2–11% at 1.25 m/s and 33–49% at 0.75 m/s (*N* = 8), alluding to 0.51–0.98 J of tendon energy returned for every 1 J stored. We interpret this finding to suggest that Direct approaches provide more plausible estimates than the Indirect approach, and may be preferable for understanding tendon energy storage and return. However, the Direct approaches did exhibit speed-dependent trends that are not consistent with isolated, *in vitro* tendon hysteresis losses of about 5–10%. These trends suggest that Direct estimates also contain some level of error, albeit much smaller than Indirect estimates. Overall, this study serves to highlight the complexity and difficulty of estimating tendon dynamics non-invasively, and the care that must be taken to interpret biological function from current ultrasound-based estimates.

## Introduction

Tendinous tissues throughout the musculoskeletal system perform a variety of important functions to enable economical, safe, and powerful movements. These tissues can serve as an energy-saving mechanism by storing and returning elastic potential energy [1–6], as a safety mechanism for muscles by absorbing energy during impact or landing [7,8], and in certain animals as a power amplification mechanism by overcoming intrinsic limitations on muscle contractile dynamics [9–11]. Tendon mechanical properties (e.g., stiffness, hysteresis loss) can be studied in isolated, *in vitro* preparations. However, the aforementioned functional roles of tendons must be studied and understood within the context of movement, highlighting the importance of *in vivo* measurement techniques able to capture tendon dynamics.

Ultrasound imaging is increasingly used to quantify tendon dynamics during human movement [12,13]. Ultrasound enables high-frequency recording of muscle fascicle and/or tendon kinematics not accessible via optical motion capture, and is beneficial for activities such as walking and running, when tendon dynamics are not feasible to study with static imaging modalities. To quantify muscle and tendon dynamics in non-human experiments, sonomicrometer crystals are often implanted to facilitate precise tracking of tissue length changes; such implants are generally not practical in human experiments. Instead, human muscle and tendon kinematics are tracked using anatomical feature tracking and a growing number of ultrasound image processing algorithms, for example, based on speckle-tracking [14] or affine optical flow [15].

**Fig 1:**
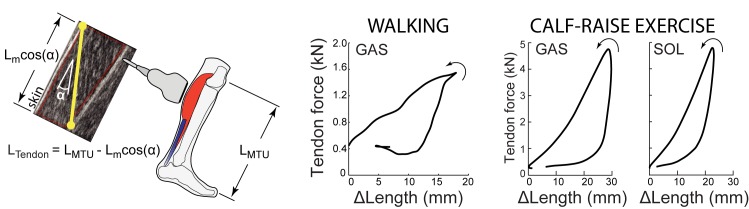
Indirect tendon estimation method and tendon work loops. Left: A common method for estimating changes in tendon length, *L*_*Tendon*_, which we term Indirect, is to subtract ultrasound-based estimates of longitudinal muscle length change, *L*_*m*_*cosα*, from overall MTU length, *L*_*MTU*_. With this method the ultrasound transducer is positioned over the muscle belly. Right: Indirect tendon kinematics can then be combined with tendon force estimates to examine tendon work loops. Walking data shown here are from [19], and calf-raise data are from [24] (see Methods for details on how data were digitized). Empirical evidence from walking (*N* = 8) [19] and from isolated calf-raise exercises (*N* = 7) [24] indicates that this Indirect method results in substantial positive work loops (i.e., net positive work performed by tendon, with 2–5 J of elastic energy returned for each 1 J stored), which is implausible for passive tendinous tissues.

Several quantitative approaches have been developed to estimate tendon kinematics from cine ultrasound images of muscle and/or tendon behavior [16]. One of the most common ways to estimate tendon kinematics, which we term *Indirect*, uses ultrasound-based measurements of muscle fascicle length change and pennation angle in conjunction with joint kinematics derived from motion capture (Fig 1). Longitudinal length changes of the muscle are subtracted from the overall muscle-tendon unit (MTU) length to estimate changes in the associated tendon length [3,17–21]. Here, MTU length is estimated from previously published regression equations based on joint kinematics; equations which themselves were derived from cadaveric studies [22,23]. This method provides a lumped estimate of tendinous tissue elongation accumulated from both the proximal and distal free tendons and aponeuroses.

Tendinous tissue length change can be combined with tendon force estimates to understand the dynamic function of tendons during movement. In prior studies, tendon force estimates have been obtained from percutaneous force transducers [19], but are more frequently obtained by dividing joint moments estimated via inverse dynamics by the tendon moment arm about the joint [17,24]. Tendon dynamics (e.g., energy storage and return) are then commonly visualized via a work loop (alternatively termed a hysteresis loop): tendon force plotted against tendon length change over a movement cycle. If net negative mechanical work is performed over a loading-unloading cycle, then this is referred to as a negative work loop, and vice versa for net positive work. For passive structures such as tendons, energy loss due to hysteresis–mechanical energy dissipated as heat during phases of material deformation and recovery–can then be estimated as the area between the tendon loading and unloading curves (i.e., the difference in energy absorbed vs. energy returned).

Validating estimates of tendon dynamics is challenging, particularly when combining tendon length change and force estimates to evaluate mechanical work and energy. This is particularly challenging for *in vivo* human studies, which generally rely on non-invasive 2D images to estimate length changes of 3D tissues, and which lack comprehensive measurement of individual muscle and tendon forces. One way to assess the validity of *in vivo* dynamics estimates is to evaluate tendon work loops. Tendinous tissues are passive (i.e., contain no active contractile elements) and viscoelastic. Thus, a simple *litmus test* (i.e., assessment in which a single factor serves as a decisive indicator) is to evaluate whether experimental estimates yield negative tendon work loops (i.e., net negative work). An ideal (lossless) spring would exhibit a net zero work loop, whereas biological tendons have been estimated to exhibit slightly negative work loops, with hysteresis losses ranging from about 5% to 30% [25–28]. Isolated *in vitro* preparations tend to exhibit values in the lower range (typically 5–10%), and *in vivo* estimates often yield estimates in the higher (10–30%) range [26,28]. Regardless of the precise magnitude of hysteresis loss in this range, we would not expect for tendons to exhibit positive work loops. After all, net positive work produced by a passive tissue would result in a negative hysteresis loss, which does not make physical sense. Net positive work would require an active, power-generating contractile element. In other words, tendon work loops derived from ultrasound imaging should at least confirm that passive, spring-like tendons are indeed behaving in passive, spring-like fashion. While not intended as a comprehensive validation, this litmus test is an important scientific sanity check, which assists us in gauging our confidence in tendon dynamics derived from ultrasound-based estimates of tendon kinematics.

There is empirical evidence that Indirect estimates of tendon kinematics, when combined with estimates of tendon kinetics, often result in large positive work loops [17,19,24,29,30], which are not physiologically plausible (Fig 1) [24]. For example, tendinous tissues have been estimated to perform 2–3 times as much positive work as negative work over the stance phase of walking (i.e., they return 2–3 J of energy for every 1 J stored), based on Indirect tendon kinematics (Fig 1, [19]). Taken at face value, this would suggest that passive tendons are acting like motors (or muscles), performing net positive work. This suggests a fundamental problem with Indirect tendon estimates, either due to measurement inaccuracy or incorrect methodological assumptions. Given the growing use of ultrasound imaging to understand the functional role of tendons during dynamic human movement and to translate this understanding, for example into the development of assistive and rehabilitative technologies, it is essential to investigate and resolve this issue.

Alternative ultrasound methods have emerged for more directly estimating tendon kinematics. A second method, which we term *Direct MTJ*, estimates tendon length changes via the position of the muscle-tendon junction (MTJ) relative to the tendon’s insertion, using motion capture-guided ultrasound imaging (i.e., transforming cine ultrasound measurements into a common reference frame with motion capture markers). A third method, which we term *Direct Tendon*, uses various speckle-tracking algorithms to quantify local elongations within the tendon [14,31–34]. However, it is currently unclear if these Direct estimates also result in physiologically implausible positive work loops, or if these yield tendon results more consistent with expected negative work loops.

The purpose of this study was to compute tendon work loops and apparent hysteresis loss using each of these Direct ultrasound estimates of tendon kinematics, and then to compare the results to previously reported values based on Indirect kinematic estimates. Although methods differ in the portion of tendinous tissue characterized, presumably with differences in absolute length change estimates, we hypothesized that tendon kinematics estimated using each Direct experimental method would yield negative work loops for the Achilles tendon during human walking across a range of speeds. We would interpret this result to be more consistent with expectations for passive tendinous tissues, thereby affirming confidence in using these estimates to understand human movement dynamics.

## Methods

### Ethical approval

The experimental protocol was approved by, and all subjects provided written informed consent according to, the University of Wisconsin Health Sciences Internal Review Board.

### Subjects and experimental protocol

We reanalyzed human walking data from our previously published work (*N* = 8, mean ± standard deviation, age: 23.9 ± 4.6 years, mass: 71.7 ± 12.8 kg, height: 1.75 ± 0.15 m) [34]. Accordingly, our cohort was not based on an a priori power analysis specific to the hypotheses presented herein. To briefly summarize, subjects walked barefoot on a dual-belt, force-sensing treadmill for 2 minutes at each of three walking speeds (0.75, 1.00, and 1.25 m/s) to precondition their Achilles tendon and allow their movement patterns to stabilize (Hawkins et al., 2009). Subjects then completed two 2-minute walking trials at each of these three walking speeds in a randomized block design, one trial for each of the two ultrasound imaging locations described below.

### Data collection

Complete measurement and analysis details are provided in Franz et al. [34], but we summarize key details below. We collected human motion and force data using standard gait analysis procedures, and employed previously-published ultrasound measurement techniques to record 2D tendon kinematics. During each trial, we collected 3D pelvis and lower-limb kinematics at 200 Hz using an optical motion capture system (Motion Analysis, Corp., Santa Rosa, CA) and treadmill ground reaction forces at 2000 Hz (Bertec, Inc., Columbus, OH). We simultaneously collected raw ultrasound radiofrequency (RF) data from a series of longitudinal cross-sections through the right plantarflexor MTU using a 10-MHz, 38-mm linear array transducer (L14-5W/38, Ultrasonix, Richmond, BC) secured using a custom orthotic. The sampling frequency for these measurements was depth-dependent. For example, for *Direct MTJ* measurements, we recorded at 128 frames/s through a 3 cm depth from a probe location centered on the distal lateral gastrocnemius (LG) MTJ. For *Direct Tendon* measurements, we recorded at 155 frames/s through a 2 cm depth from a probe on the distal free Achilles tendon, centered on average 6 cm superior to the posterior calcaneus marker. The image resolution for all RF recordings was 128 × 1560 pixels (e.g., 0.297 mm × 0.013 mm pixel size for Direct Tendon). We collected the position and orientation of the ultrasound transducer using three retroreflective markers placed on the custom orthotic. For each of these two imaging locations, we recorded 5 strides per condition distributed over each 2 minute walking trial. We later identified these strides in our motion capture data. Using an analog sync signal emitted from the Ultrasonix system, the ultrasound and motion data were synchronized to within 5 ms (i.e., temporal resolution of the 200 Hz motion capture system).

### Data analysis

**Fig 2:**
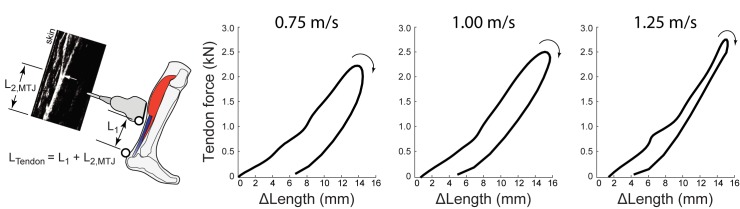
Direct MTJ estimation method and tendon work loops during walking. Left: The Direct MTJ method estimates tendon length changes by summing the instantaneous position of the ultrasound transducer with respect to the tendon’s insertion, *L*_1_, and the displacement of the MTJ within the transducer’s field of view, *L*_2,*MTJ*_. In this method the ultrasound transducer is positioned over the MTJ. Right: This Direct MTJ method, applied to the LG MTU, yields negative work loops on average, for all walking speeds tested. Hysteresis losses were estimated to decrease with increasing speed, from an average of 49% at 0.75 m/s to 11% at 1.25m/s. Average (inter-subject) mean results are depicted (*N* = 8).

**Fig 3:**
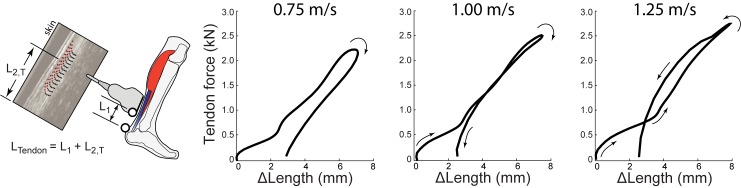
Direct Tendon estimation method and tendon work loops during walking. Left: The Direct Tendon method estimates tendon length changes by summing the instantaneous position of the ultrasound transducer with respect to the tendon’s insertion, *L*_1_, and the average local displacements of tendon tissue within the transducer’s field of view, *L*_2,*T*_, based on speckle- tracking analysis. In this method the ultrasound transducer is positioned over the Achilles free tendon, distal to the soleus MTJ. Right: This Direct Tendon method yields negative tendon work loops on average, for all walking speeds tested. Similar to trends observed in the Direct MTJ results, hysteresis losses estimated from the Direct Tendon method were also observed to decrease with increasing walking speed, from an average of 33% at 0.75 m/s to 2% at 1.25 m/s. Average (inter-subject) mean results are depicted (*N* = 8).

We analyzed each series of ultrasound data in two phases. Phase I consisted of estimating local tissue displacements from the ultrasound recordings (i.e., displacements within the probe’s field of view). The Direct MTJ method used custom software implemented in MATLAB (Mathworks, Inc., Natick, MA) to manually track the local displacements of the distal LG MTJ in B-mode images created from the RF data, similar to previous studies using this estimation method (e.g., [20,35]). The Direct Tendon method used a 2D ultrasound speckle-tracking algorithm to track longitudinal free Achilles tendon tissue displacements based on previously published and validated techniques [36,37]. To summarize, for each corresponding walking trial, we manually positioned a ~15 mm x 3 mm grid of nodes (1 mm × 0.5 mm spacing) comprising a rectangular region of interest on a B-mode image of the minimally-loaded Achilles tendon at the instant of toe-off. The region of interest width ensured that only tendinous tissue was included in the tracking routine. To track free Achilles tendon tissue motion, we calculated two-dimensional cross-correlation functions between upsampled (4x) RF data within a 2 mm × 1 mm kernel surrounding each nodal position and a 2.8 mm × 1.4 mm search window in each successive frame. Peaks of these frame-to-frame cross-correlations defined the gross nodal displacements which were refined by estimating subpixel displacements using the peaks of a 2D quartic spline surface fit to the correlation functions. Final nodal trajectories were determined as the weighted average of forward and backward tracking results assuming cyclic motion trajectories. For the present analysis, we used the average of all nodal trajectories as a gross estimate of Achilles tendon tissue displacement over a walking stride. Phase II: We defined Achilles tendon elongations by co-registering local displacements of the LG MTJ (Direct MTJ method, Fig 2) and the Achilles Free tendon (Direct Tendon method, Fig 3) with the instantaneous, interpolated position of the posterior calcaneus marker, a surrogate for the tendon’s insertion, using coordination transformations established previously [34].

We calculated net ankle moment via standard inverse dynamics analysis and used this to approximate Achilles tendon force, as detailed below. Marker trajectories and ground reaction force measurements were fourth-order low-pass filtered at 6 Hz and 100 Hz cutoff frequencies, respectively. We assumed that the net ankle extensor moment was borne completely by forces generated by the triceps surae (soleus, medial and lateral gastrocnemius) muscles and transmitted through the Achilles free tendon. Accordingly, we calculated the instantaneous Achilles tendon force by dividing the net ankle moment by subject-specific measures of the Achilles tendon moment arm. We estimated this moment arm during walking as the average perpendicular distance between the tendon’s line of action, registered in ultrasound images of the free Achilles tendon, and the transmalleolar midpoint as a surrogate for ankle joint center [38].

Combining estimates of tendon kinematics (from ultrasound) and kinetics (from inverse dynamics), we then calculated stance phase tendon work loops (tendon force versus tendon length change), derived from both Direct MTJ and Direct Tendon approaches. Next, we integrated these tendon work loops to calculate stance phase positive work (during tendon loading, in Joules) and negative work (during unloading). Net work was then computed by summing negative work (energy absorbed during tendon loading) and positive work (energy returned during tendon unloading). Net work provides an estimate of the hysteresis loss of the tendon. However, to facilitate comparisons between walking speeds we divided this value by the negative work (tendon energy stored) and report hysteresis loss as a percentage, a common convention in biomechanics (e.g., [26]). Net tendon work and hysteresis loss from Direct MTJ vs. Direct Tendon approaches were compared statistically using a two-way (method × walking speed) repeated measures ANOVA with an alpha value of 0.05. We also tested whether hysteresis loss differed significantly from 0% using one-sample t-tests.

### Data digitization from prior literature

Hysteresis loss was estimated from previously published studies that computed tendon kinematics via the Indirect method, and which also reported an estimate of tendon force. Several previous studies have presented figures showing experimentally-estimated Achilles tendon work loops (e.g., [24]), or plots of tendon force and tendon displacement over time (e.g., [19]). In both cases these are sufficient data to compute tendon hysteresis loss; however, hysteresis losses were not explicitly computed nor reported in these articles. We therefore used freely available data digitization software to extract work loop and/or time-series data from the pertinent figures in each publication. Data digitization involves: (i) defining the directions of x and y axes on the figure, (ii) calibrating these axes using numerical axis labels in the publication to convert between physical distances on the page and actual units of tendon force or length, (iii) manually tracing each data curve by selecting discrete points along it to capture a series of x and y data points, then (iv) exporting these data points (in units of force or length based on the established calibration) for post-processing. From Sakuma et al. ([24], their Fig 4, *N* = 7) we extracted work loop data from calf-raise bouncing, specifically tendon force in Newtons and length in mm. These data were imported into Matlab, where we integrated the work loops to calculate positive and negative work, then used these work values to compute tendon hysteresis loss (identical to calculations performed for Direct MTJ and Direct Tendon methods). From Ishikawa et al. ([19], their Fig 2, *N* = 8) we extracted Achilles tendon force (kN) vs. time (% contact), as well as tendon length change (%) vs. time (% contact) waveforms for walking. The time axis in both plots was identical and synchronous. Digitized force and length curves were each resampled to 200 data points (representing 0 to 100% of stance phase). We then plotted tendon work loops (force vs. length). From this plot we computed tendon hysteresis loss, as detailed above. Note that because the hysteresis loss computed is a unitless quantity (reflecting the percentage of energy loss over a loading-unloading cycle), work loops can be plotted in arbitrary units. In other words, hysteresis loss (in %) is independent of whether force is plot in units of N or kN, or tendon length change is plotted in mm or as a percentage. Finally, tendon work loops and hysteresis losses from our own study (estimated via Direct MTJ and Direct Tendon approaches) were qualitatively compared to those extracted from prior literature (estimated via the Indirect method). To maintain consistent figure axes, we report all forces in kN and all length changes in mm.

## Results

### Indirect tendon estimation techniques

Based on digitized data from previously published studies, tendon length changes estimated from measured muscle fascicle kinematics consistently produced positive tendon work loops and thus apparent negative hysteresis loss, indicative of considerable but physiologically implausible positive work generation. During isolated calf-raise bouncing exercises [24], tendon hysteresis loss derived from soleus fascicle kinematics was approximately -200%, signifying about 3 J of tendon energy return for every 1 J of tendon energy stored (Fig 1). Hysteresis losses derived from gastrocnemius fascicle kinematics were even larger in magnitude, at least -400%. During walking [19], tendon hysteresis loss derived from soleus and gastrocnemius fascicle kinematics elicited values of approximately -130% and -200%, respectively.

### Direct tendon estimation techniques

Across the range of speeds tested, peak Achilles tendon force during the stance phase averaged 2.2–2.7 kN. This range of peak forces was associated with tendon length changes of 14.3–15.2 mm estimated via LG Direct MTJ and 7.1–7.8 mm estimated via Direct Tendon approaches. In contrast to Indirect estimates, we found that both Direct techniques yielded, on average, negative tendon work loops and thus positive percentages for tendon hysteresis loss during the stance phase of walking. Tendon hysteresis loss (and net work) estimated using Direct MTJ averaged 48.5 ± 16.9% (-9.0 ± 6.3 J), 37.3 ± 27.1% (-8.3 ± 6.9 J), and 11.3 ± 38.6% (-5.2 ± 9.5 J) for walking at 0.75, 1.00, and 1.25 m/s, respectively (Fig 2). Hysteresis loss differed significantly from 0% at 0.75 m/s (p<0.01) and 1.00 m/s (p<0.01) for Direct MTJ estimates. Tendon hysteresis loss estimated using Direct Tendon averaged 32.9 ± 26.4% (-3.4 ± 3.3 J), 11.0 ± 33.2% (-2.0 ± 3.9 J), and 2.1 ± 33.5% (-1.2 ± 3.9 J), respectively (Fig 3). Hysteresis loss differed significantly from 0% at 0.75 m/s (p = 0.02) for Direct Tendon estimates. Net tendon work (main effect, p = 0.04), but not hysteresis loss (main effect, p = 0.34), differed significantly between the two Direct measurement techniques. Finally, both outcome measures decreased significantly and progressively with increasing walking speed (main effect, p<0.01).

## Discussion

Ultrasound imaging is increasingly used to quantify tendon dynamics during movement in humans and other animals. Frequently, these tendon dynamics are estimated from Indirect measures of muscle kinematics, but there is mounting evidence that this approach consistently yields implausible tendon work loops. More direct estimates of tendon dynamics are also possible, either via tracking MTJ kinematics or localized tendon kinematics. In contrast to Indirect estimates, and in support of our hypothesis, we found that both Direct estimates of tendon kinematics generally resulted in negative work loops during human walking, with hysteresis losses averaging 2–49% across the walking speeds tested. In other words, for every 1 J of energy stored elastically by tendon, Direct estimates would suggest that 0.51–0.98 J of this energy was returned. In contrast, based on hysteresis losses derived from Indirect tendon kinematics estimates, for every 1 J of energy stored elastically by tendon, 2–5 J of energy would be returned. As discussed earlier, it is implausible for a passive tendon to return 100–400% more energy than it stores. Therefore, we conclude that compared to Indirect estimates, Direct ultrasound estimates of energy storage and return are more plausible, and thus may be preferable for understanding tendon dynamics during human movement.

Our results provide important benchmark data for net tendon work and hysteresis loss during human locomotion assessed via two emerging Direct ultrasound estimates, relative to previously reported Indirect estimates. Unsurprisingly, Direct MTJ work magnitudes were systematically larger than Direct Tendon estimates, given the differences in probe placement (Fig 2 vs. Fig 3), which ensure that Direct MTJ captures kinematics from a larger portion of the tendon. At 1.25 m/s, hysteresis loss from both Direct kinematics estimates (2–11%, on average) was reasonably consistent with previously reported tendon hysteresis loss measured *in vitro* [26]. However, the Direct tendon hysteresis loss magnitudes at slower speeds (e.g., >30% at 0.75 m/s) were unusually large compared to values obtained from isolated tissue preparations, generally <10% [28]. Furthermore, the Direct kinematics estimates exhibited unexpected speed-dependent hysteresis loss trends (Figs 2 and 3), which are not consistent with what is known about isolated tendon behavior. Although tendon loading rate may increase with gait speed, mechanical testing of cadaver tendons indicates that increased loading rates (within the ranges that occur during locomotion) have minimal effect on hysteresis loss (e.g., [39,40]). Thus Direct estimates also appear to contain some level of hysteresis loss error, albeit much smaller than Indirect estimates. As such, Direct estimates and their observed speed-dependent hysteresis loss warrant further study, and still require cautious interpretation.

The reason that these Direct estimates yielded larger hysteresis losses at slower speed is currently unclear, but might be resolvable through modification of the present methodologies. These refinements may include, in part, relaxing the assumption of a linear tendon path between the ultrasound probe and calcaneus [41], improving the tendon force estimate, and/or accounting for load-dependent moment arm characteristics [38]. We also observed that for a small subset of individual subjects walking at 1.25 m/s, even the Direct estimates occasionally yielded positive work loops (the worst-case subject exhibited apparent hysteresis loss of -45%). Studying faster walking speeds and/or larger sample sizes may be needed to elucidate the source and prevalence of inter-subject variability. Overall, this study serves to highlight the complexity and difficulty of estimating tendon dynamics non-invasively, and the care that must be taken to interpret biological function from current tendon estimates. Further research is greatly needed to ascertain how to more faithfully estimate and interpret tendon dynamics during functional movement.

It remains unclear which fundamental assumptions or measurement inaccuracies result in the substantial positive tendon work loops obtained from Indirect tendon kinematics estimates. While the focus of this study was on quantifying human tendon dynamics *in vivo*, it is notable to mention that positive tendon work loops from Indirect estimates have also been observed in data from animal studies [42], highlighting the broader importance of understanding and resolving this issue. Below we summarize several potential culprits. First, MTU lengths based on regression equations from historical cadaver data may not be sufficiently representative of individual subjects, and thus could introduce significant errors. Second, the assumption that the aponeurosis acts longitudinally and in series with muscles and tendons neglects potentially important dynamics in the transverse plane [43], which may lead to an overestimation of positive work performed by passive viscoelastic tissues. Researchers have previously presented both analytical arguments [44] and empirical evidence [42] for why “the assumption that the aponeurosis is in series with the tendon must be either completely abandoned or, at least, justified as a valid approximation in each particular case,” ultimately concluding that “tendon, aponeurosis, and muscle fiber forces are related in a complex and highly non-intuitive way. Any simplistic modeling or interpretation of the muscle-tendon system, any interpretation of aponeurosis strain in relation to tendon force, or any considerations of elastic force recovery in aponeurosis segments using tendon force may produce incorrect results and interpretations of the mechanics and energetics of muscle contraction” [44]. Third, muscle fascicle length changes derived from 2D images may not accurately reflect the presumably complex 3D motion of MTUs. Muscle contractions can induce substantial bulging (i.e., 3D shape change) effects, and depending on factors such as probe placement and orientation, the longitudinal tendon length change estimates may fail to capture transverse loading dynamics, particularly of the aponeurosis [43,45,46]. Energy storage due to force and displacement along unmeasured (transverse) dimensions could conceivably be returned longitudinally along the tendon, resulting in the appearance of net positive tendon work from the current ultrasound estimation methods, since these only capture longitudinal dynamics. Fourth, the tendon moment arm estimate could also introduce error in tendon force estimates. MTU moment arms are generally treated in biomechanical analysis as either a constant value or solely dependent on joint kinematics; however, recent evidence suggests that moment arms are also affected by other factors such as muscle loading [38] and muscle geometry [47]. Fifth, tendon force estimates, often computed indirectly from net ankle moments derived from inverse dynamics, could be (and likely are) somewhat inaccurate due to the various assumptions underlying these analyses [48]. Examples of confounding factors include the fact that a small amount of force is also borne through other (non-triceps surae) muscles that cross the ankle joint [49], and that the Achilles tendon is comprised of fascicles originating from each of the triceps surae muscles which introduces more complicated, non-uniform loads and displacements [34]. Nevertheless, it is unlikely that uncertainty in tendon force estimates fully explain the positive tendon work loops from Indirect kinematics, since these same force estimates were also used in conjunction with Direct tendon kinematics estimates in this study, and this did not result in positive tendon work loops on average. Also, one of the previous studies employing Indirect tendon length change estimates [19] used a percutaneous force sensor (not inverse dynamics) and still found substantial positive tendon work loops.

Further studies are needed to identify which of these, or other, methodological issues underlie the implausible hysteresis losses derived from Indirect tendon kinematics. In the meantime, interpreting absolute magnitudes of tendon energy storage and return from Indirect tendon kinematics should be avoided; particularly when physiologically implausible net positive tendon work is estimated. In this study, we highlight Indirect tendon estimation issues in human movement data, but most of the problems enumerated above are also potentially problematic when studying tendons in other species, potentially even if sonomicrometer crystals are implanted to more precisely track muscle length changes.

This study highlights the benefits of simple litmus tests for investigating *in vivo* human biomechanics, a field fraught with imperfect and incomplete measurements due to the immense complexity of the musculoskeletal system. Such litmus tests, founded in classical mechanics, have been successfully applied in other aspects of biomechanics to evaluate and improve methodology; for instance, to assess the completeness of inverse dynamics joint work [50–52]. With regards to quantifying tendon dynamics, the work loop litmus test could be evaluated for a variety of tasks (beyond level ground walking), to further assess confidence in tendon dynamics estimates. The litmus test detailed in this study should not be interpreted as a complete validation of any methodology; however, it provides a useful and non-invasive way to assess the plausibility of results. In contrast to animal or human cadaver studies, comprehensive ground truth data (e.g., tendon force and strain from implanted sensors) are generally impractical or unethical (e.g., cutting the Achilles tendon to insert a load cell in series) to obtain during *in vivo* human experiments. This precludes more traditional means of validating estimates against ground truth, meaning it is generally not possible to place a definitive numerical value on the accuracy of a given *in vivo* method. Such quantification of accuracy can often only be obtained in animal or cadaver studies, but then one must assume that this same level of accuracy is also present in human subject experiments. Ultimately, the validity of *in vivo* tendon estimates can be assessed through an accrual of evidence from multiple partial validation tests, such as the litmus test discussed in this study. Additional experiments (both *in vivo* and *in vitro*), computational analyses, and theoretical approaches are needed to provide complementary validation and a more comprehensive understanding of measurement capabilities and limitations.

Several study limitations are worth acknowledging. This study focused solely on Achilles tendon behavior during level ground walking. It would be beneficial to apply similar analyses to other tendinous tissues and additional tasks with altered biomechanical demands (e.g., incline/decline gait) [53], to further explore whether work loop estimates are consistent with expectations for passive elastic tissues. In terms of evaluating Indirect tendon kinematics, we relied on published findings and did not recollect those data for analysis in this study. Based on the availability of published literature using this Indirect method (which reported time-varying Achilles tendon force and synchronized estimates of tendon length change), we were not able to precisely match the walking speeds that we analyzed via Direct methods. Presented in Fig 1 are results from walking at 1.4 m/s [19], which was the closest we found to our top speed of 1.25 m/s. Other published walking studies we found either reported higher speeds (e.g., [17]), or reported muscle activity instead of estimated Achilles tendon force (e.g., [20]). Regardless of the precise gait speed, we found that multiple publications from different research groups using this Indirect kinematics approach have reported considerable net positive tendon work, for both walking and other tasks [17,19,24,29,30]. Thus, this issue appears to be potentially ubiquitous for the methodology. It is conceivable that for certain movement tasks, or by altering certain methodological assumptions, the Indirect approach may yield more physiologically plausible work loops. However, we have not found clear evidence of this in the published literature. In this study, the tendon force estimate was derived by dividing ankle moment (from inverse dynamics) by the average Achilles tendon moment arm. This estimate assumes a rigid foot, an invariant moment arm, and that the net plantarflexion moment was borne by force through the Achilles tendon. Finally, we note that our ultrasound measurements were synchronized to within 5 ms of the motion and force data. Based on a previously published sensitivity analysis [26], ±5 ms would be expected to introduce up to ±5% uncertainty in our hysteresis loss estimates. Such errors would not alter our conclusions in this study.

This study focuses on the accuracy and physiological plausibility of tendon dynamics estimates, as opposed to the precision of estimates. Accuracy (closeness of a measurement to its true value) is important for certain subsets of scientific questions (e.g., quantifying the absolute magnitude of energy storage and return by tendons). Precision (closeness of repeated measurements to each other) is important for other types of questions (e.g., when comparing relative changes between conditions). In some cases, absolute accuracy may be less important and indeed may not be essential for testing hypotheses or reaching robust scientific conclusions. As such, this study should not be read as an indictment of the rich body of literature that has employed Indirect (or Direct) means of characterizing trends or relative changes in muscle-tendon dynamics. In fact, the method in this study termed Indirect (in relation to capturing tendon kinematics) still remains the preeminent way of quantifying muscle fascicle kinematics *in vivo* during movement, and its usefulness should not be underappreciated for understanding muscle dynamics. Each measurement method and modality provides a snapshot of one component of the musculoskeletal system, and data obtained are combined using simplifying assumptions to approximate and understand complex biological movement. The challenge moving forward is not simply to identify which method is better, but rather to discover how to optimally combine experimental kinematics and kinetics to answer targeted questions related to muscle and/or tendon function. Proper data fusion relies not only on the placement of the ultrasound probe and features tracked, but also on our conceptual framework for analyzing and interpreting results. As with any simplified model/method, we must work to understand if/when our underlying assumptions regarding muscle-tendon behavior provide reliable estimates and when they begin to breakdown.

## Conclusion

In conclusion, as we advance our scientific understanding of movement biomechanics, it is important to continue advancing and validating our experimental methods. *In vivo* imaging modalities such as ultrasound offer unique opportunities to explore muscle-tendon dynamics in humans and other animals, but as with all biomechanical measurements these techniques have limitations. The accuracy and completeness of our biomechanical estimates affect our scientific interpretations as well as our applied interventions. Our results are highly relevant to the degree to which tendon elastic energy storage and return facilitate economical locomotion, and to the bio-inspired design and prescription of assistive devices that seek to restore/augment human calf muscle-tendon function. Musculoskeletal simulations also rely on accurate empirical biomechanical estimates, either as direct inputs or indirectly for validation. It can be difficult to reconcile implausible experimental estimates (e.g., considerable positive work performed by tendons) with modeled dynamics (e.g., tendon passivity constraints). Ultimately, we must understand the accuracy, precision, benefits, drawbacks, and assumptions of each measurement approach in order to appropriately interpret the functional role of muscle-tendon dynamics during movement.

## Supporting information

S1 File Subject-specific data used to create work loop figures and compute results. (ZIP)

## Abbreviations

LG: lateral gastrocnemius
MTJ: muscle-tendon junction
MTU: muscle-tendon unit
