# Supplementary material for: It’s positive to be negative: Achilles tendon work loops during human locomotion
Source: PLoS One. 2017 Jul 3;12(7):e0179976. doi: 10.1371/journal.pone.0179976 (PMC5495204; doi:10.1371/journal.pone.0179976)
Supplement: S1 File — (ZIP) [file pone.0179976.s001.zip › S1_File/Electronic Supplementary Materials - File List.pdf]

Electronic Supplementary Materials – File List

Subject-specific data used to create work loop figures and results in the manuscript are provided in the following CSV files. Measurement units are denoted in square brackets. Each column in the CSV file represents mean data from a single subject. Sagittal plane ankle moment and tendon length change data represent 0 to 100% of a stride, where 0% corresponds to toe-off (i.e., the beginning of swing phase).

**Filename**	**Description**	**Units**
Data from Direct MTJ trials		
direct_mtj_ankle_moments_0p75.csv	Sagittal plane ankle moments for each subject at 0.75 m/s	[Nm/kg]
direct_mtj_ankle_moments_1p00.csv	Sagittal plane ankle moments for each subject at 1.00 m/s	[Nm/kg]
direct_mtj_ankle_moments_1p25.csv	Sagittal plane ankle moments for each subject at 1.25 m/s	[Nm/kg]
direct_mtj_tendon_length_0p75.csv	Tendon length change based on Direct MTJ method, at 0.75 m/s	[mm]
direct_mtj_tendon_length_1p00.csv	Tendon length change based on Direct MTJ method, at 1.00 m/s	[mm]
direct_mtj_tendon_length_1p25.csv	Tendon length change based on Direct MTJ method, at 1.25 m/s	[mm]
Data from Direct Tendon trials		
direct_tendon_ankle_moments_0p75.csv	Sagittal plane ankle moments for each subject at 0.75 m/s	[Nm/kg]
direct_tendon_ankle_moments_1p00.csv	Sagittal plane ankle moments for each subject at 1.00 m/s	[Nm/kg]
direct_tendon_ankle_moments_1p25.csv	Sagittal plane ankle moments for each subject at 1.25 m/s	[Nm/kg]
direct_tendon_tendon_length_0p75.csv	Tendon length change based on Direct Tendon method, at 0.75 m/s	[mm]
direct_tendon_tendon_length_1p00.csv	Tendon length change based on Direct Tendon method, at 1.00 m/s	[mm]
direct_tendon_tendon_length_1p25.csv	Tendon length change based on Direct Tendon method, at 1.25 m/s	[mm]
Anthropometric data pertinent to all trials		
moment_arms.csv	Achilles tendon moment arm about the ankle joint (sagittal plane)	[m]
